# Tumor mutational burden presents limiting effects on predicting the efficacy of immune checkpoint inhibitors and prognostic assessment in adrenocortical carcinoma

**DOI:** 10.1186/s12902-022-01017-3

**Published:** 2022-05-14

**Authors:** Fangshi Xu, Yibing Guan, Peng Zhang, Li Xue, Yubo Ma, Mei Gao, Tie Chong, Bin-Cheng Ren

**Affiliations:** 1 Department of Medicine, Xi’an Jiaotong University, No. 76, Yanta West Road, Xi’an, 710061 Shaanxi China; 2 Department of Urology, The Second Affiliated Hospital of Xi’an Jiaotong University, No. 157, West Five Road, Xi’an, 710000 Shaanxi Province China; 3 Department of Rheumatology and Immunology, The Second Affiliated Hospital of Xi’an Jiaotong University, No. 157, West Five Road, Xi’an, 710000 Shaanxi Province China

**Keywords:** Tumor mutational burden, Adrenocortical carcinoma, Immune checkpoint inhibitors, Immune microenvironment, Prognosis

## Abstract

**Background:**

Adrenocortical carcinoma (ACC) is a highly malignant urologic cancer and tends to metastasize. Although immune checkpoint inhibitors (ICIs) bring a glimmer of light to conquer ACC, only a fraction of patients have benefit from ICIs treatment. It is well known that tumor mutational burden (TMB) is closely associated with the efficacy and response rate of immunotherapy. However, its roles in ACC were not investigated.

**Methods:**

Using somatic mutations data of 92 ACC samples in TCGA database, we calculated their TMB values by the ‘maftools’ package in R software (Ver 3.6.3). To explore the roles of TMB in ICIs therapy, we have addressed this issue from three perspectives. First, the effects of TMB levels on tumor immune microenvironment (TIM) were analyzed through CIBERSORT algorithm, ssGSEA method and TIMER web server. Second, we investigated the expressive correlations between TMB level and five pivotal immune checkpoints based on Pearson coefficient. Third, the difference in TIDE score between high- and low-TMB groups was compared. The prognostic value of TMB was also evaluated. Besides, GSEA was performed to determine the changes in the activities of signaling pathways caused by TMB.

**Results:**

TMB values in ACC samples were not high. The average of total mutation counts in each sample was only 21.5. High TMB could lead metabolic reprogramming and poor survival outcomes. However, it was unable to affect the infiltration levels of lymphocytes, and failed to facilitate the activities of immune-related pathways. Regarding immune checkpoints (ICs), only PD-L1 upregulation could result in a good prognosis, and TMB level did not correlate with the expressions of other ICs except for LAG3. There was no significant difference in TIDE score between high- and low-TMB groups. Combining the present results and previous study, we speculated that inadequate stimulation for neoantigens formation, intrinsic immune-resistance and special genomic alterations were three possible reasons for TMB limiting functions in TIM and ICIs. Besides, TMB was toughly applied in clinical practice due to its high cost of determination and non-universal definition of high TMB.

**Conclusions:**

TMB presents limiting effects on prediction for ICIs efficacy and prognostic assessment for ACC patients.

## Background

Adrenocortical carcinoma (ACC) is a highly malignant urologic cancer and tends to metastasize. The overall survival (OS) time of ACC patients is commonly less than 30 months, while the 5-year overall survival rate (OSR) of patients with clinical stage III-IV is only 13% [1]. Although surgery is the most effective therapeutic approach, the resectable cases only account for 16% in all ACC patients [2]. Even after complete surgical resection, approximately 40% of patients will progress to metastatic disease within 2 year [3]. As the basic adjuvant therapy for advanced ACC, the response rate of mitotane plus chemotherapy was less than 30% [4]. Thus, the treatment of ACC is extremely challenging.

Recently, immune checkpoint inhibitors (ICIs) lead a significant breakthrough in cancer treatment and bring a glimmer of light to conquer ACC. Since 2016, five agents targeting the programmed death-1/ligand-1 (PD-1/L1) have been approved to treat urothelial carcinoma, especially for metastatic cancer [5]. However, only a fraction of patients can present a good therapeutic response. A Phase 2 study revealed that, for ACC patients treated with pembrolizumab, their clinical benefit rate reached up to 54%, but their objective response rate (ORR) was just 15% [6]. Therefore, it is urgent and meaningful to identify an effective predictive biomarker for the efficacy of ICIs.

Tumor mutational burden (TMB) refers to the totality of somatic mutations per million bases, including single nucleotide polymorphisms (SNPs) and variations of copy number. High number of mutations in somatic exonic regions commonly induces the formation of neoantigen, which activates T cell immunogenicity to inhibit tumor cells and brings a better therapeutic response [7]. Hence, TMB is regarded as a potential biomarker for predicting the efficacy and response rate of immunotherapy [7]. In the phase III CheckMate 227 study, nivolumab plus ipilimumab significantly prolonged progression-free survival (PFS) of non-small-cell lung cancer (NSCLC) patients with high TMB (≥10 mutations per megabase, MB) comparing with chemotherapy [8]. Given that cancer patients with high TMB commonly have a higher response rate to anti-PD-1/L-1 immunotherapy, the US Food and Drug Administration (FDA) even have approved pembrolizumab for patients with malignant solid tumors of any histologic type with high TMB (≥10 MB) in June 2020 [9]. However, the roles of TMB in ACC has not been fully elucidated. In the present study, we investigated the relationships between TMB, immune microenvironment and ICIs therapy in ACC using bioinformatics method, and found a dramatically different outcome. High TMB may not affect the ACC immune microenvironment and not serve as a predictive biomarker for ICIs efficacy.

## Materials and methods

### Data source

The analytical data were obtained from the Cancer Genome Atlas (TCGA) public database (https://portal.gdc.cancer.gov/repository). There were three data categories were downloaded, namely gene expression data (240 ACC samples), somatic mutations data (92 ACC samples) and clinical data (90 ACC samples). The data type of gene expression was ‘Transcriptome profiling’, and that of somatic mutations was ‘Simple nucleotide variation’. The workflow type of somatic mutations was chosen as ‘VarScan2 Annotation’. The data format of clinical data was ‘BCR-XML’. The clinical features of 90 ACC samples were shown in Table 1.

**Table 1:** Clinical characteristics of 90 ACC patients from TCGA database

Variables	Number (percentage)
Vital status	
Alive	57 (63.3%)
Dead	33 (36.7%)
Age	NA
Gender	
Male	30 (33.3%)
Female	60 (66.7%)
Tumor Grade	NA
Clinical Stage	
Stage I	9 (10.0%)
Stage II	44 (48.9%)
Stage III	19 (21.1%)
Stage IV	18 (20.0%)
T stage	
T1	9 (10.0%)
T2	49 (54.4%)
T3	11 (12.2%)
T4	21 (23.4%)
M stage	NA
N stage	
N0	80 (88.9%)
N1	10 (11.1%)

*ACC* Adrenocortical carcinoma, *NA* not available

### The calculation of TMB value

The visualization of somatic mutations data was performed via the ‘maftools’ package in R software (Ver 3.6.3) [10]. The concurrent and exclusive associations across mutated genes were determined by using ‘corrplot’ package in R software. TMB represents the total number of mutations per megabyte in tumor samples, which was defined as the average number of somatic mutations in tumor genome, including gene coding errors, base substitution insertions and deletions [11]. Due to the length of human exon being commonly set as 38 MB (million bases), the TMB value of each ACC sample was calculated as the total mutation frequency/38 [12].

### GSEA

Gene-set enrichment analysis (GSEA) is a computational method that determines whether an a priori defined set of genes shows statistically significant and concordant differences between two biological phenotypes [13]. In the present study, GSEA was performed to compare the differences in signal pathways between different TMB levels. Enrichment statistics was based on weighted method. Phenotype labels were set as high TMB level versus low TMB level. Permutation Type was phenotype. The number of permutations was set as 1000, and ‘c2.cp. Kegg.v7.4 symbols’ was chosen as the gene set. When the normalized enrichment score (NES) ≥1, nominal (NOM) *p*-value ≤0.05 and false discovery rate (FDR) *q*-value ≤0.25 were simultaneously satisfied, the pathways were considered to significantly enriched.

### Survival analysis and clinical correlation analysis

According to the median of TMB value, 90 ACC samples were divided into high- and low-TMB groups. The ‘survival’ package in R software was employed to analyze the prognostic differences between high- and low-TMB groups. Receiver operating characteristic curve (ROC) was applied to assess the predictive accuracy of TMB. Cox univariate and multivariate analyses were successively conducted to identify whether TMB was an independent prognostic factor of ACC. The relationships between TMB and clinicopathological features of ACC were determined based on Kolmogorov–Smirnov test.

### Immune analysis

CIBERSORT algorithm can estimate the abundances of various kinds of human leukocyte subtypes through the gene expression data [14]. This method is based on deconvolution algorithm, and relies on a LM22 reference set which provides a set of gene expression features of 22 leukocyte subtypes [15]. 1000 permutations and *p*-value<0.05 were set during CIBERSORT analysis. Then, the immune abundances of 22 leukocyte subtypes in each ACC sample were calculated and their visualizations were displayed through ‘barplot’ package in R software. To evaluate the effect of TMB on ACC immune microenvironment, the difference in infiltration levels of multiple immune cells between high- and low-TMB groups was compared. Its visualization was performed via ‘vioplot’ package. Moreover, the active scores of 13 immune-related pathways were calculated based on single-sample gene set enrichment analysis (ssGSEA).

TIMER web server is a comprehensive resource for systematical analysis of immune infiltrates across diverse cancer types. The ‘Survival’ module was applied to confirm the associations of infiltration levels of immune cells with survival outcomes in ACC [16].

### Correlation analysis of immune checkpoints and TMB

To explore the relationships between TMB and the efficacy of ICIs, we demonstrated this issue in two ways. First, we analyzed the expressive correlations between TMB level and five pivotal immune checkpoints (ICs) (including PD-L1, CTLA4, LAG3, HAVCR2 and TIGIT), which was quantized by Pearson coefficient. Besides, the differences in the expressions of five ICs between high- and low-TMB levels were calculated through ‘ggplot2’ and ‘reshape2’ R packages.

Second, we also employed TIDE score to shed light on this issue. In 2018, Jiang P’s team developed a novel algorithm, tumor immune dysfunction and exclusion system (TIDE), to predict the outcome of cancer patients treated with PD-1/L1 inhibitors [17]. The higher TIDE score commonly indicates a greater propensity for dysfunction of T cells, therefore leading a worse efficacy of PD-1/L1 inhibitors. Therefore, we compared the difference in TIDE score between high- and low-TMB levels via ‘Limma’ R package.

## Results

### TMB level in ACC is not high

**Fig. 1:**
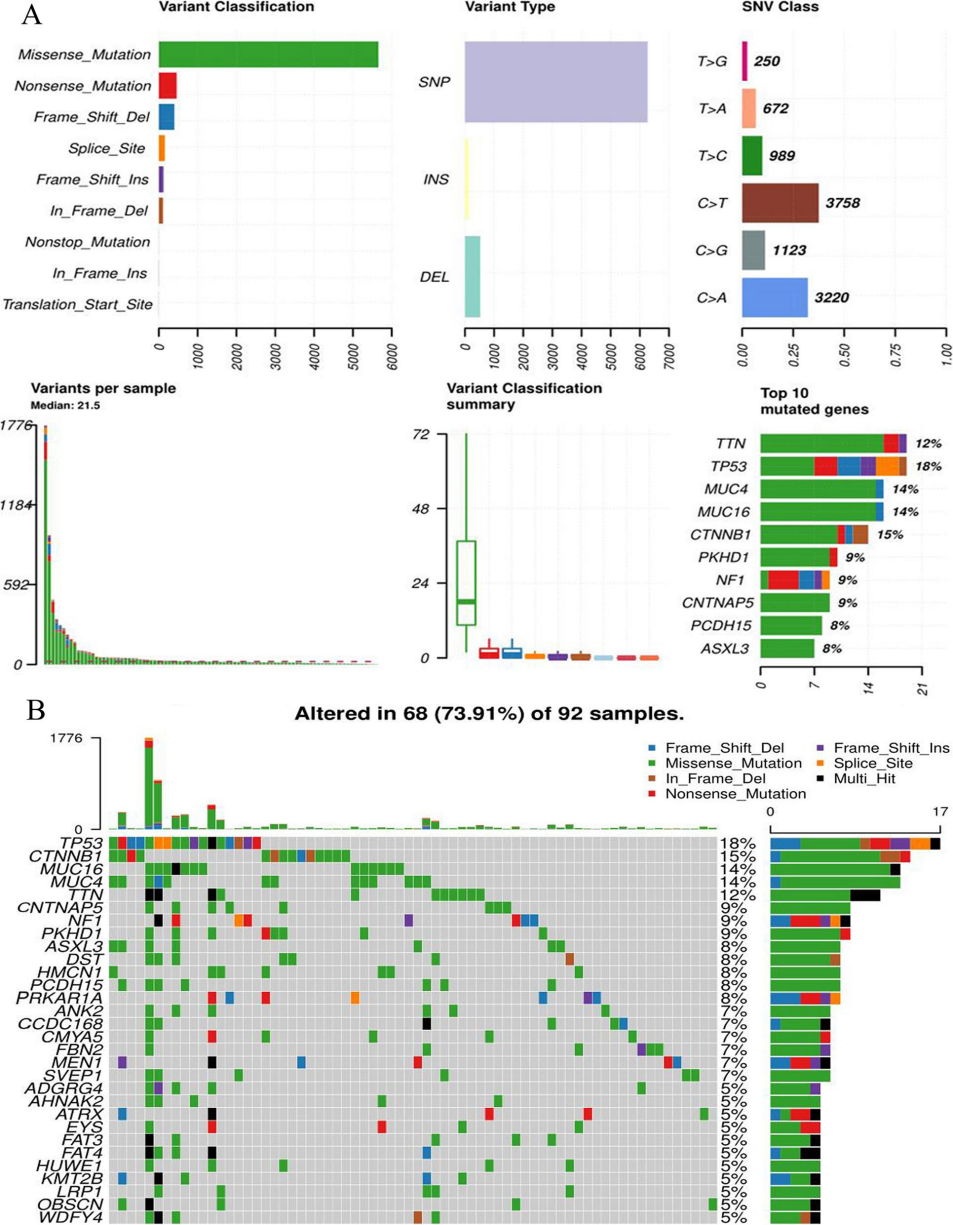
The landscape of somatic mutations in ACC. **A** The summary of mutational type. **B** The waterfall plot of top 20 mutated genes. ACC, adrenocortical carcinoma; INS, insertion; DEL, deletion; SNV, single nucleotide variant; SNP, single nucleotide polymorphism

Using 92 ACC samples in TCGA database, we exhibited the landscape of somatic mutations in ACC (Fig. 1). Missense mutation was the most common mutational form. Single nucleotide polymorphism (SNP) accounts for the largest part of all variant types (Fig. 1A). C>T and C>A transitions were both major classes of single nucleotide variants (SNV) (Fig. 1A). In addition, the waterfall plot demonstrated the profile of somatic mutation information in each ACC sample (Fig. 1B). Somatic mutations occurred in 73.91% of all ACC samples. However, the average of total mutation counts in each sample was only 21.5 (Fig. 1A), and the highest gene mutation frequency was just 18% (TP53) (Fig. 1B). These results indicated that although somatic mutations frequently occurred in ACC samples, the mutation burden was not high.

### Alterations of TMB levels lead metabolic reprogramming in ACC

**Fig. 2:**
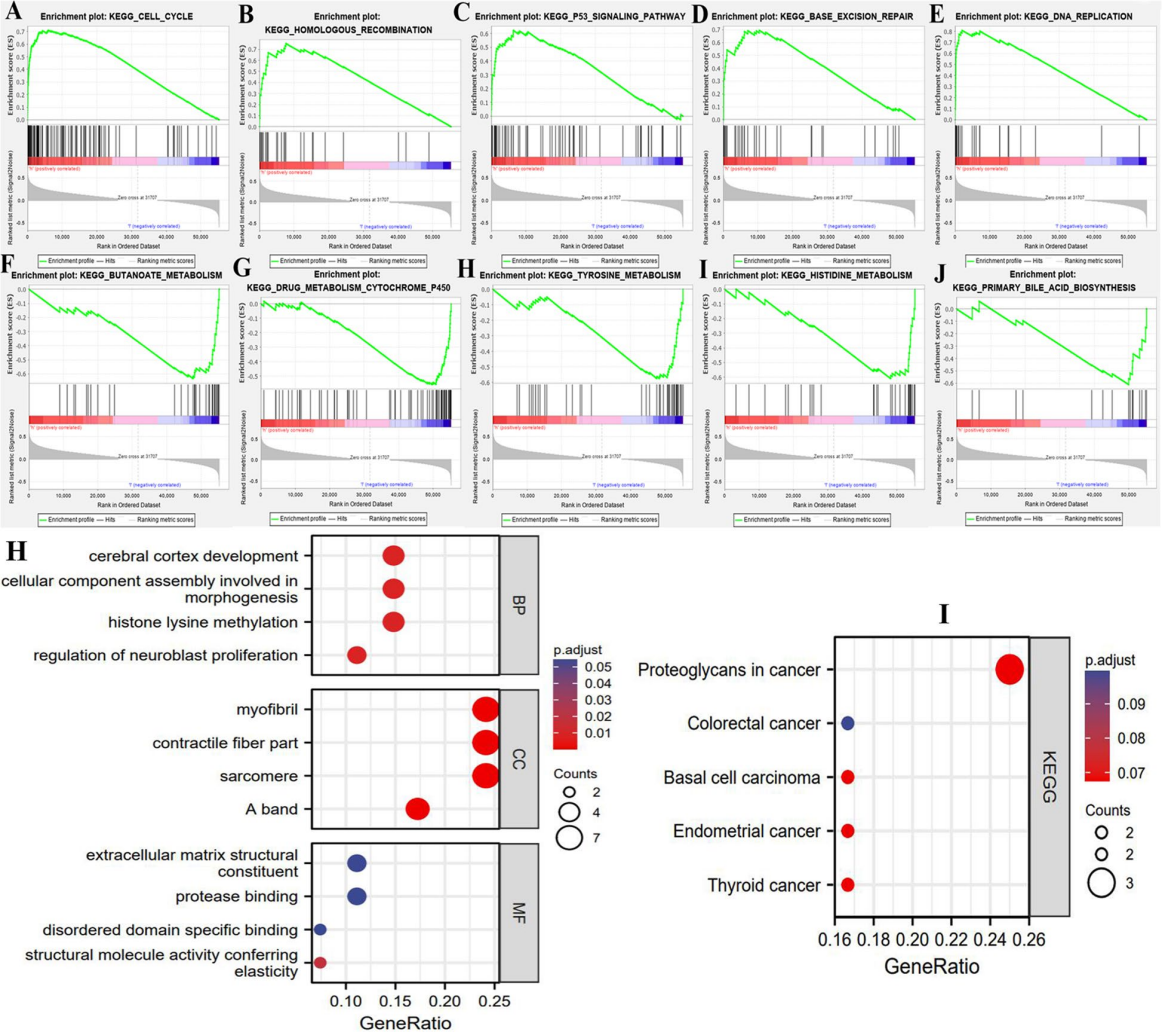
Changes in TMB levels lead metabolic reprogramming in ACC. **A**-**E** The top 5 enriched signaling pathways in high-TMB group. **F**-**J** The top 5 enriched signaling pathways in low-TMB group. **H** The functional enrichment analyses of 30 most frequently mutated genes in ACC. **I** The KEGG analyses of 30 most frequently mutated genes in ACC. TMB, tumor mutational burden; BP, biological process; CC, cellular component; MF, molecular function; KEGG, Kyoto encyclopedia of genes and genomes

The GSEA results indicated that different TMB levels had a dramatic impact on the activities of signaling pathways (Fig. 2A-J). In high TMB group, cell proliferation-related pathways, including ‘cell cycle’, ‘Base excision repair’ and ‘DNA replication’ pathways were significantly enriched, which revealed that high TMB could promote ACC progression (Fig. 2ADE). Nevertheless, the enrichments of ‘homologous recombination’ and ‘p53’ pathways caused by high TMB would bring an inhibitory effect on malignant progression. As shown in Table 2, the deficiency of homologous recombination can impede the process of DNA double-strand break repair, which commonly promote tumorigenesis (Fig. 2B) [18]. p53, a famous tumor suppressor protein, exerts its anti-tumor functions through promoting apoptosis and DNA repair [19]. Hence, its enrichment is unfavorable for cancer development.

**Table 2:** The effect of TMB level on signaling pathways

Pathway	Variation trend	NOM *p*-value	Functions in cancer
Homologous recombination	Enriched in high TMB	0.008	Main steps in DNA double-strand break repair.
P53	Enriched in high TMB	0.000	Tumor suppressor protein.
Butanoate metabolism	Enriched in low TMB	0.017	1. An anticancer ingredient. 2. Butanoate metabolism is disrupted in breast cancer.
Tyrosine metabolism	Enriched in low TMB level	0.008	Tyrosine kinase family encompasses the greatest number of oncoproteins.
Histidine metabolism	Enriched in low TMB level	0.028	Histidine metabolism can increase the effectiveness of methotrexate treatment.
Bile acid metabolism	Enriched in low TMB level	0.035	Bile acids have been proposed to promote colon carcinogenesis.
Cytochrome P450	Enriched in low TMB level	0.002	Cytochrome P450 enzymes play key roles in bile acid synthesis.

*TMB* tumor mutational burden

Metabolic reprogramming is a major hallmark of cancer. In ACC, we found that five metabolic signaling pathways enriched in low-TMB group (Table 2). Among that, butanoate has served as an anticancer ingredient [20], and its metabolism pathway has been found to be disrupted in breast cancer patients [21]. Therefore, butanoate metabolism has been regarded as an approach to differentiate cancer patients from healthy individuals [21]. Histidine metabolism can boost cancer therapy through increasing the effectiveness of methotrexate treatment [22]. Bile metabolism has been proven to regulate tumorigenesis, and high level of bile acid could promote colon proliferation [23, 24]. In a word, the alterations of TMB levels lead metabolic reprogramming in ACC, which may be involved in the regulation of ACC progression.

Besides, we investigated the biological functions of 30 most frequently mutated genes in ACC samples, including TP53, CTNNB1, MUC16, MUC4 and TTN etc. (Fig. 1B). These genes were closely involved in the proliferation- and invasion-related process, such as ‘cellular component assembly involved in morphogenesis’, ‘extracellular matrix structural constituent’ and ‘structural molecule activity conferring elasticity’ (Fig. 2H). For instance, extracellular matrix (ECM) is the dynamic foundation of tumorigenesis and cancer progression [25]. The active assemblage of ECM undoubtedly contributes to cancer development. Meanwhile, these mutated genes also profoundly affected the regulation of multiple cancers (Fig. 2I). Briefly, frequently mutated genes in ACC were tightly associated with tumor malignant behavior.

### High TMB results in poor prognosis and unfavorable clinical stages

**Fig. 3:**
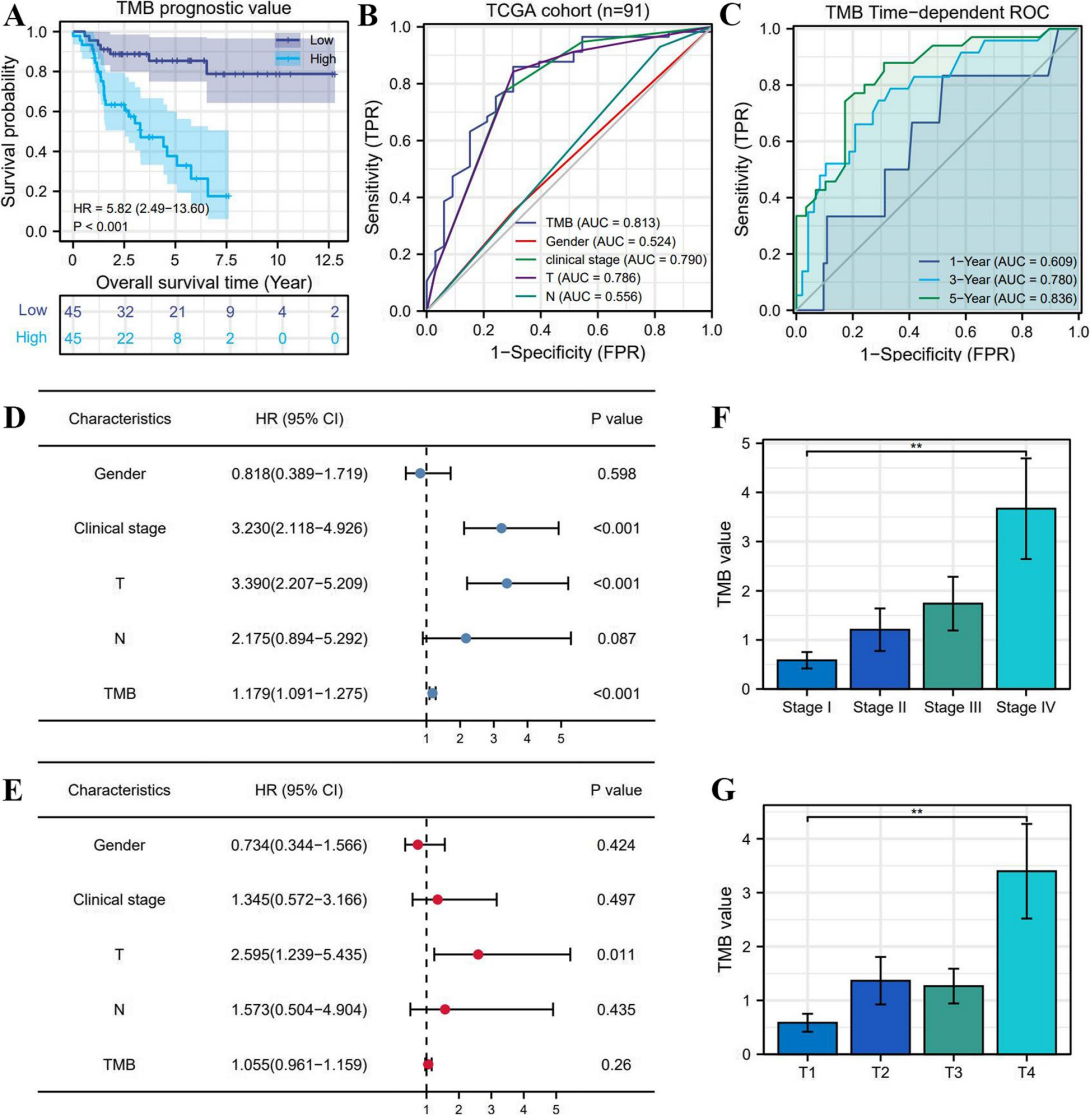
The prognostic value of TMB in ACC. **A** The prognostic difference between high- and low-TMB groups. **B**, **C** The prediction accuracy of TMB. **D** Univariate independent prognostic analysis. **E** Multivariate independent prognostic analysis. **F**, **G** High TMB is correlated with advanced clinical and T stages

According to the median of TMB (2.19/MB), 90 ACC samples were classified into high- and low-TMB groups. The prognostic analysis revealed that high TMB conferred an adverse survival outcome (HR = 5.82, *P*<0.001) (Fig. 3A). The 5-year OSRs of high- and low-TMB groups were 34.7 and 82%, respectively. Meanwhile, the TMB was found to have a preponderance of prognostic prediction over other clinical indicators (Area under curve, AUC = 0.813) (Fig. 3 BC). Interestingly, despite the great predicting performance of TMB, it was not an independent prognostic factor of ACC (HR = 1.055, *P* = 0.26) (Fig. 3DE). In addition, High TMB was significantly associated with advanced clinical stage (Fig. 2F) and T stage (Fig. 2G).

### High TMB cannot affect the immune microenvironment of ACC

**Fig. 4:**
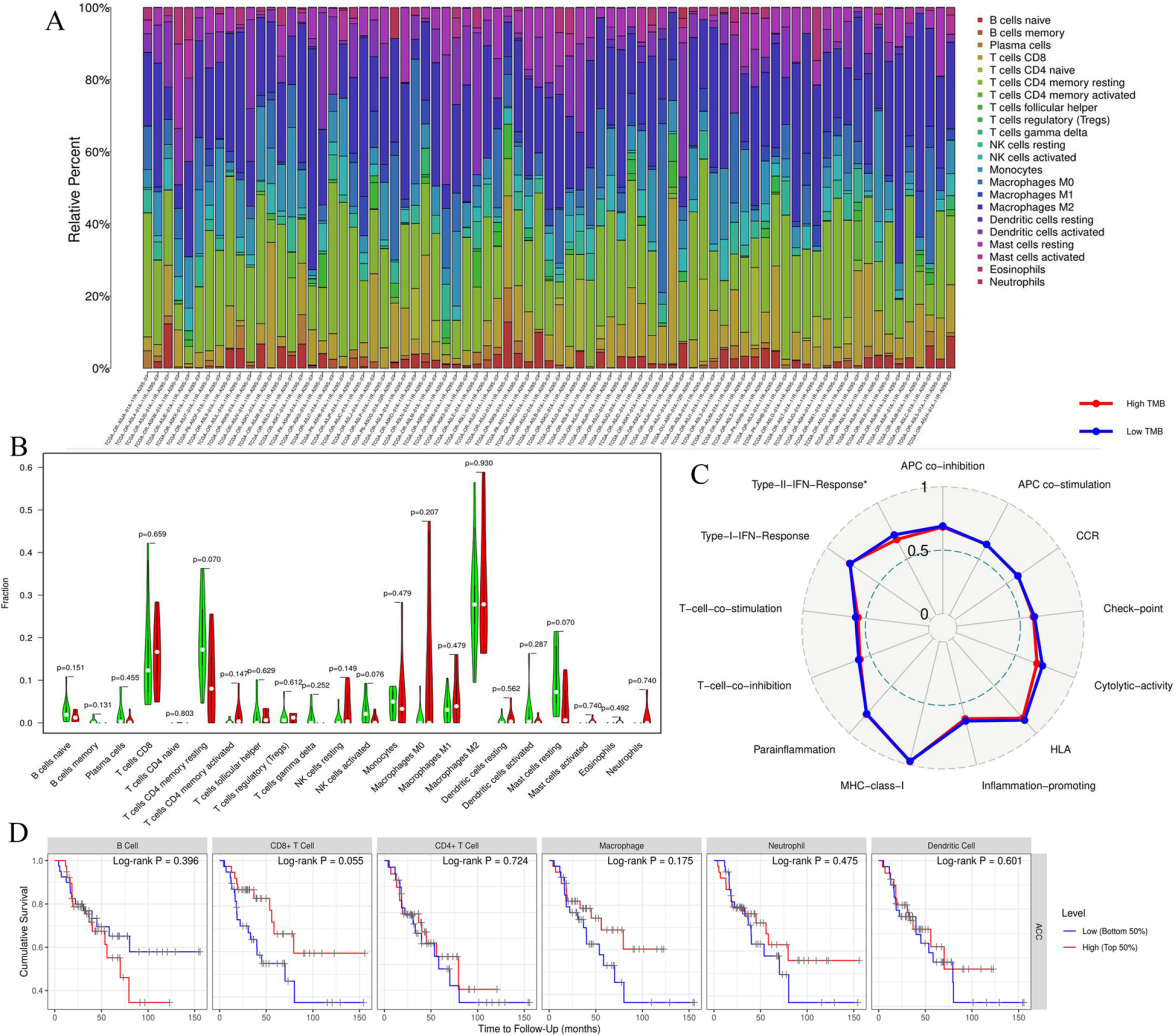
TMB cannot affect ACC immune microenvironment. **A** The landscape of immune abundance of 22 leukocyte subtypes in each ACC sample. **B** Comparison of the infiltrating levels of 22 immune cells between different TMB groups. Green means low-TMB group; Red means high-TMB group. **C** Comparison of the activity scores of 13 immune-related pathways between different TMB groups. **D** The effects of six key immune cells on ACC prognosis

Using the CIBERSORT algorithm, the immune abundances of 22 leukocyte subtypes in each ACC sample were exhibited in Fig. 4A. Interestingly, high TMB not only cannot affect the infiltration levels of lymphocytes (Fig. 4B), but also cannot facilitate the activities of immune-related pathways (Fig. 4C). Particularly, as the pivotal immune cells for targeting cancer, the immune abundances of CD8+ T, CD4+ T and NK cells did not present significantly differences between high- and low-TMB groups (Fig. 4B). Moreover, the infiltration levels of six hub immune cells also could not affect ACC prognosis (Fig. 4D), which suggested that ACC may equip with a certain degree of immune resistance.

There were no observable differences in the activities of 13 immune-related pathways between different TMB levels (Fig. 4C). It is well known that ICIs therapy is tightly related to ‘antigen-presenting process’ [26], ‘immune checkpoint expression’ [27] and ‘cytolytic effect’ [28]. Nevertheless, these immune pathways all failed to be affected by high TMB (Fig. 3C).

### TMB may not serve as a biomarker for predicting the efficacy of ICIs

**Fig. 5:**
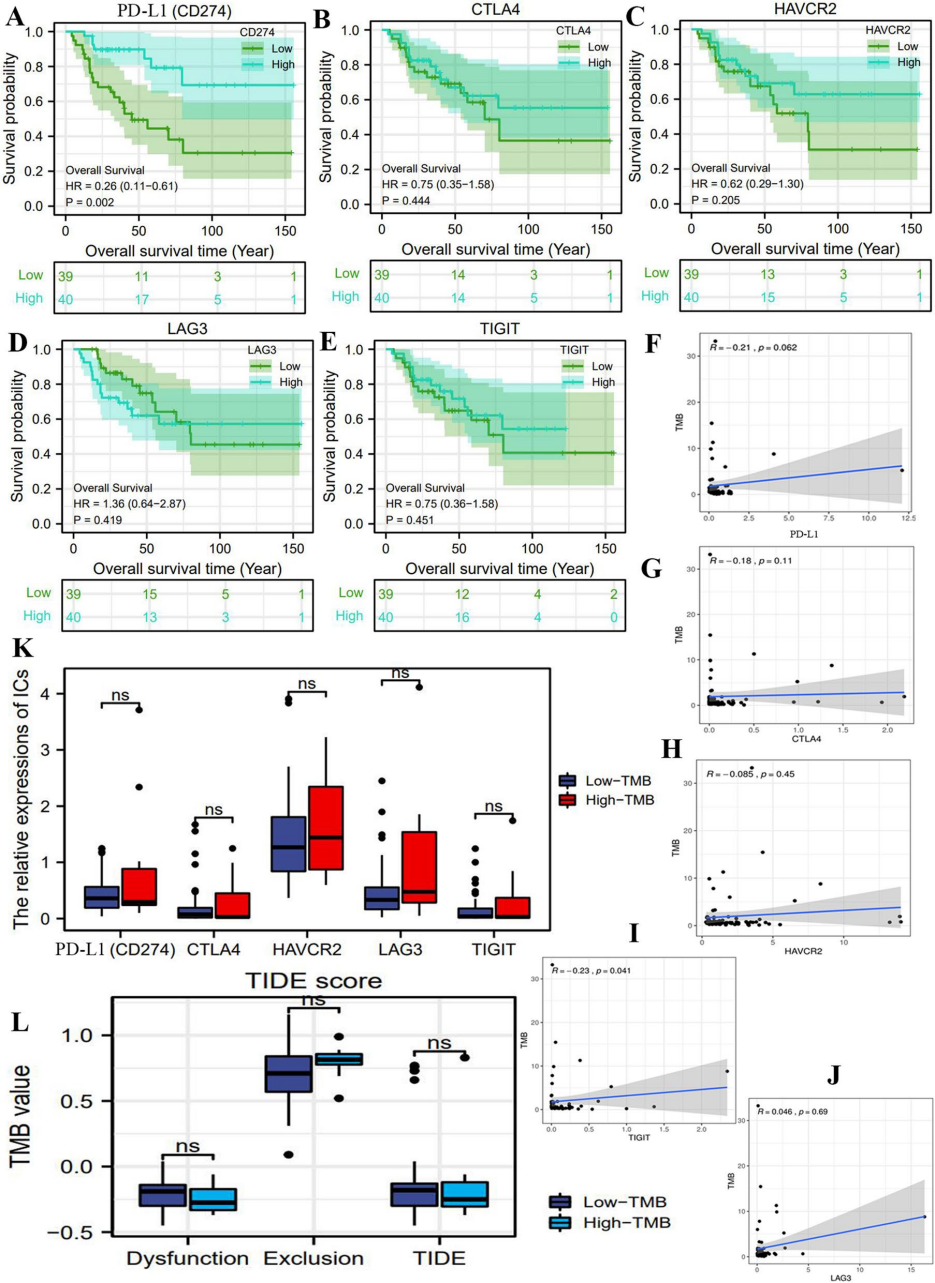
TMB may not serve as a biomarker for predicting the efficacy of ICIs. **A**-**E** The effects of five immune checkpoints on ACC prognosis. **F**-**J** The expressive correlations between TMB and immune checkpoints. **K** The expressive differences of immune checkpoints between high- and low-TMB groups. **L** The difference in TIDE score between high- and low-TMB groups

In the present study, we investigated the potential linkages between TMB and ICIs therapy from three aspects. First, the impacts of immune checkpoints (ICs) on ACC prognosis. Overexpression of ICs in tumor cells is implicated in immune exemption and immune escape through suppressing the functions of T cells [29], which commonly will lead an adverse prognosis for cancer patients. Through survival analyses, we found that only PD-L1(CD274) upregulation could result in a good prognosis (Fig. 5A), whereas other ICs were unable to affect the survival outcomes of ACC patients (Fig. 5B-E). Similarly, Billon et al. also confirmed that high expression of PD-L1 was a favorable prognostic marker in ACC [30]. These findings indicated that the changes in ICs expressions was weakly related to ACC prognosis, therefore targeting ICs therapy was not obviously beneficial for ACC patients.

Second, although appointing a reliable marker for predicting ICIs efficacy is not conclusive, the patients with PD-1/L1 positive expression commonly presented a better therapeutic response [31]. Except for LAG3, TMB level did not correlate with the expressions of PD-L1, CTLA4, HAVCR2 and TIGIT (Fig. 5F-J). Furthermore, the expressive levels of all ICs in high TMB group was comparable to those in low TMB group (Fig. 5K).

Third, TIDE score can quantify the possibility that patients will benefit from anti-PD-1/L1 or anti-CTLA4 treatment [17]. Nevertheless, there was no significant difference in TIDE score between high- and low-TMB groups (Fig. 5L). This observation revealed that TMB level was not competent to play as a biomarker for predicting the efficacy of anti-PD-1/L1 therapy.

## Discussion

**Fig. 6:**
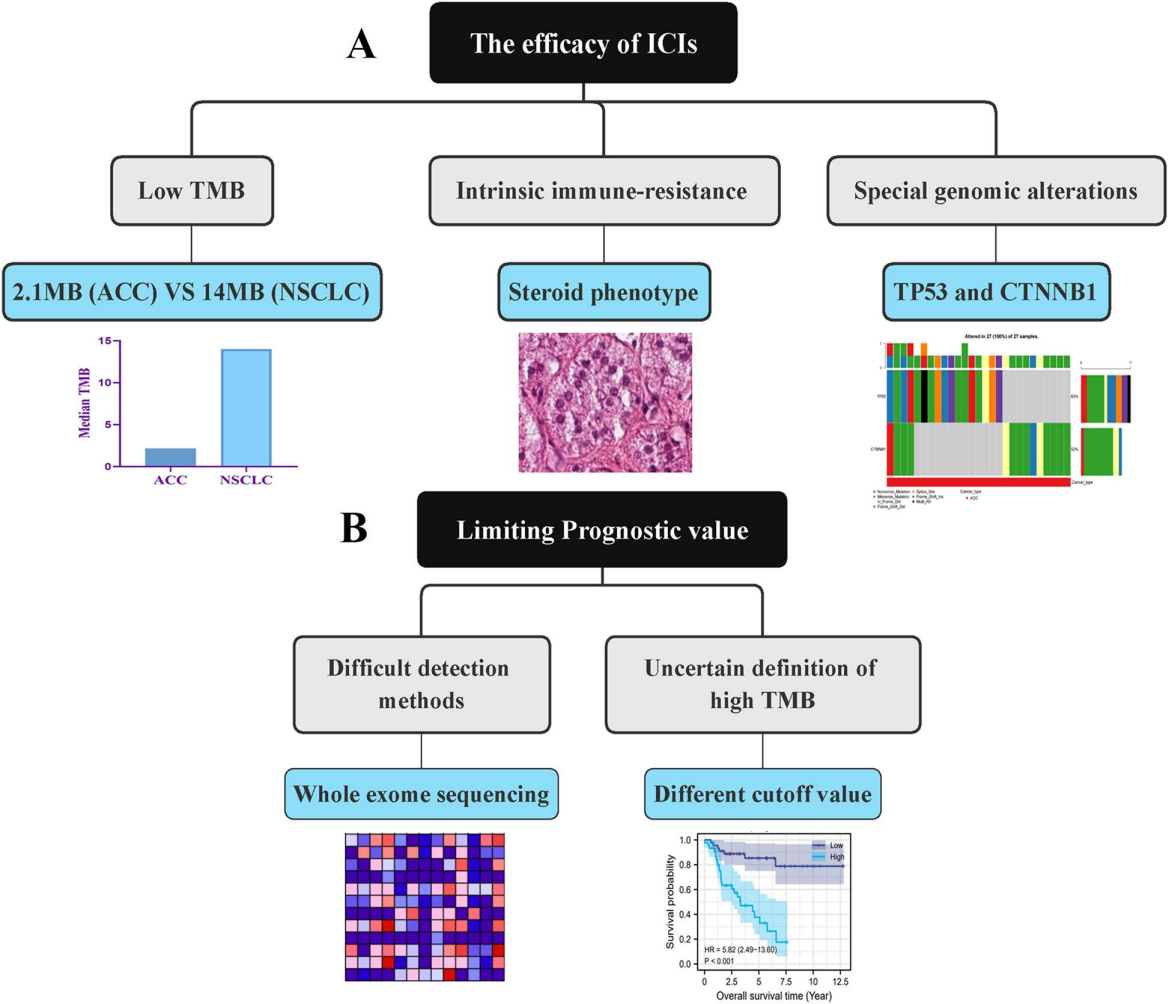
TMB has limiting effects on predicting the efficacy of ICIs and prognostic assessment in ACC. TMB, tumor mutational burden; ACC, adrenocortical carcinoma; ICIs, immune checkpoints inhibitors; NSCLC, non-small-cell lung cancer

ACC is highly malignant and tends to metastasize. Neither surgical excision nor mitotane cannot achieve a satisfactory therapeutic effect. In recent years, immune checkpoint inhibitors (ICIs) provides a promising option for metastatic ACC, and have been initially applied in urothelial carcinoma [5]. However, only a small proportion of patients can benefit from ICIs therapy. TMB is considered as a potential biomarker for predicting ICIs efficacy [32], and has been validated in lung cancer and hepatocellular carcinoma [8, 33]. Nonetheless, in the present study, we found that TMB has limiting effects on predicting ICIs efficacy and prognostic assessment in ACC through preliminary bioinformatic analyses (Fig. 6AB).

### Why TMB is not conducive to predicting the efficacy of ICIs in ACC?

Immune checkpoint, especially the overexpression of PD-1/L1 and CTLA4 can generate inhibitory signals of T-cell function, thus suppressing anti-tumor immune process. From the perspective mechanisms, CTLA4 has higher avidity and affinity to ligands B7–1 and B7–2 so that it can competitively inhibit molecule T-cell receptor (TCR) signaling [34]. As for PD-1/L1, it is capable of attenuating T-cell activation through transmitting the tyrosine phosphatase SHP2 [34]. In consequence, the expression status of immune checkpoint is strongly associated with efficacy of ICIs [35]. Although it is so far inconclusive about identifying a reliable biomarker for predicting the efficacy of ICIs, many clinical trials have reported that PD-1/L1 positive expression commonly implied a better response to ICIs. For instance, in NCT01693562 trial referring to metastatic urothelial carcinoma (UC), the objective response rate (ORR) in PD-L1 positive patients was up to 46.4%, by contrast, that in PD-L1 negative patients was even 0% [36]. Given that the therapeutic advantage brought by PD-1/L1 overexpression, many researchers supported PD-1/L1 expression as a predictive biomarker for ICIs response [31]. Moreover, it is now well recognized that tumor immune microenvironment (TIM) has a great influence on tumor progression and response to immunotherapy [37]. For example, as the pivotal immune cells for retarding cancer, dysfunction of CD8+ T cells will promote immunologic tolerance, thereby induce immunotherapy resistance [38]. The activation of CD8+ T cells requires the assistances of antigen-presenting cells (APCs) and CD4+ T cells. Conversely, macrophage M2 cells and regulatory T cells (Tregs) can make immunologic barriers against the process [38]. Therefore, difference in TIM will create the difference  in response to ICIs treatment.

In the present study, we speculated that TMB was weakly associated with ICIs therapy based on threefold findings. First, TMB could not lead any changes in infiltration levels of immune cells in ACC. Patients with high- or low-TMB seem to possess similar TIM. Meanwhile, some ICIs-related pathways such as ‘APC process’, ‘check-point’ and ‘cytolytic activity’ could not be motivated by high TMB level (Fig. 4C). Second, there was no expressive correlations between TMB and immune checkpoints (Fig. 5F-K). TMB level was not capable of affecting the expressive status of ICs. Third, TIDE score indicated that there were no significant differences in the occurrence probabilities of immune exclusion and T cells dysfunction between high- and low-TMB groups (Fig. 5L). These findings reiterated that ACC patients with high TMB could not benefit from ICIs therapy. Naturally, TMB may not serve as an effective biomarker for predicting ICIs efficacy. Referring to some prior research, we hypothesized that there were three possible reasons for the interesting phenomenon (Fig. 6A).

First, the level of TMB in ACC is too low to propel the formation of neoantigens. As acknowledged, the accumulation of TMB induces the production of neoantigens, thus stimulating the activity of T cell and promoting anti-tumor immune effect. Nevertheless, not all mutations result in immunogenic neoantigens and determining which somatic mutations are likely to induce neoantigens remains extremely challenging. Therefore, only a certain level of TMB accumulation can trigger the formation of neoantigens. Therefore, TMB represents a quantifiable approach to measure the probability of neoantigen production [39]. The higher TMB, the greater chance to produce immunogenic neoantigens. Hellmann MD et al. found that PFS of patients receiving nivolumab plus ipilimumab with a high TMB (≥10 MB) was significantly longer than that of patients receiving conventional chemotherapy (42.6% versus 13.2%) [8]. Based on POPLAR (NCT01903993) and OAK (NCT02008227) trials, Gandara DR et al. defined the high TMB as larger than 14 MB and regarded high TMB as a predictor of clinical benefit in NSCLC patients with atezolizumab treatment [40, 41]. By contrast, in our study, the median of TMB in ACC was only 2.1 MB, which was far lower than that in lung cancer (10 or 14 MB). As a result, we speculated that low accumulation of TMB in ACC was not enough to induce the formation of tumor neoantigens, therefore, it cannot affect the immune microenvironment of ACC. Second, ACC is provided with intrinsic immune-resistance. It has been proven that ACC patients with cortisol secreting usually accompanied with the suppression of T cell activity [42]. This subtype of ACC, so called steroid phenotype, commonly displays the low pathological immune scores, revealing its immune-resistance [42]. Meanwhile, tumor-related glucocorticoid excess occurs in about 60% of all ACC patients, which also cause the depletion of T cells and undesirable prognosis [42]. Therefore, the negligible impact of TMB on immune microenvironment of ACC may result from the immune-resistance of ACC itself. Third, some characteristic genomic alterations of ACC also mediated the immunological tolerance in ACC. In the present study, TP53 and CTNNB1 were the most mutated genes (Fig. 1B). Ragazzon B et al. confirmed that 52% of invasive ACC patients possessed the mutations of TP53 or CTNNB1, suggesting that the mutations of two genes were involved in the malignant progression of ACC [43]. More importantly, TP53 mutation can lead the decreased lymphocytic infiltration levels and thereby induce the occurrence of tumor immune escape. In addition, overexpression of CTTNB1 was also proven to reduce the infiltration levels of tumor-infiltrating lymphocytes (TILs) and CD8+ T cells, and was associated with immunosuppression and poor prognosis in ACC [44]. Therefore, the frequently mutated TP53 and CNNB1 in ACC also bring a suppression on infiltration levels of immune cells and retard the anti-tumor immunological process. This may be the genomic element that makes TMB fail to affect immune microenvironment of ACC.

### Why TMB is not helpful to prognostic assessment in ACC?

Except for predicting ICIs efficacy, TMB has been found to strongly associated with cancer prognosis. Nevertheless, TMB presents different prognostic characteristics in different cancers. It was a poor prognostic marker for neuroblastoma [45] and glioma [46], but a good prognostic marker for NSCLC [47]. Meanwhile, Hwang, W. L et al. argued that TMB was beneficial to optimize prognosis stratification and guide treatment decisions [45]. Although we also found that high TMB led to a poor survival outcome (Fig. 3A), we still thought that the suggestive effect of TMB on prognostic assessment was fairly limiting at the present stage. First, the determination of TMB value needs to perform the whole exome sequencing (WES) on tumour DNA and matching normal DNA [48]. High costs and substantial turnaround time for analysis and sequencing are obviously not applicable to routine detection and screening. Second, we lacked one universal definition of high TMB. A pan-cancer research revealed that a specific TMB cutoff can be always found to distinguish the prognosis of patients for most cancers, but the specific cutoff points markedly varied across different cancer types [49]. Altogether, due to the difficult detection process and the inconsistent standards of high TMB, it fails to effectively to contribute to the prognostic assessment of ACC (Fig. 6B).

### The mutations of titin and mucin do not seem to work in ACC

Due to the large size of exon, the mutations of titin (TTN) and mucin (MUC) are frequently detected in multiple solid tumors, and closely correlated with TMB and the objective response to ICIs [50]. For example, MUC16 was mutated in 38.4% of gastric cancer (GC) patients, and was associated with higher TMB and good immune response [51]. Therefore, Li X et al. considered that its mutation could guide immunotherapy treatment for GC patients [51]. Besides, a pan-cancer research revealed that a positive correlation was observed between the ORR of ICIs therapy and the frequency of TTN mutation, which also heralded that high TTN mutation predicted a better response rate to ICIs [50]. However, although TTN (12%), MUC4 (14%) and MUC16 (14%) commonly mutated in ACC (Fig. 1B), these mutations did not seem to be correlated with the efficacy of ICIs for ACC cases. There are two possible reasons to make such phenomenon.

First, the mutation frequency of MUC16 in ACC (14%) was much lower than that in other solid tumors, such as GC (38.4%), lung adenocarcinoma (42.76%) and melanoma (73.86%) [52]. This directly leads that low mutation of MUC16 is not competent to bring a higher TMB to trigger a strong immune response [49]. Second, the roles of MUC16 and TTN mutations in the anti-tumor immune process were still unclear. For example, MUC16 could hinder the targeting of NK cells to tumor cells [53]. Curiously, MUC16 mutations was commonly accompanied with better prognosis and therapeutic response [51]. The underlying mechanisms remain elusive.

### Limitations and conclusions

Naturally, there are some limitations in our research. First, the conclusion of this study is based on bioinformatics analyses and has not been verified by real clinical cohorts. Second, insufficient ACC data in TCGA database may lead biased conclusions.

Collectively, according to our findings, TMB may not be appropriate to predict ICIs efficacy in ACC. Inadequate stimulation for neoantigens formation, intrinsic immune-resistance and special genomic alterations were three possible reasons. Moreover, although TMB could affect the survival outcomes of ACC patients, it was toughly applied in clinical practice because of its high cost of determination and non-universal definition of high TMB. In a word, TMB presents limiting effects on predicting ICIs efficacy and prognostic assessment for ACC patients. Our findings provided an interesting insight into ACC immunotherapy and prognosis analyses.

## Data Availability

The datasets used and/or analyzed in the current study are available from the corresponding author upon reasonable request.

## Abbreviations

ACC: Adrenocortical carcinoma
TMB: Tumor mutational burden
ICIs: Immune checkpoint inhibitors
PD-1: Programmed death-1
CTLA4: Cytotoxic T-Lymphocyte-Associated Antigen 4
SNPs: Single nucleotide polymorphisms
PFS: Progression-free survival
NSCLC: Non-small-cell lung cancer
MB: Mutations per megabase
SNV: Single nucleotide variant
TIM: Tumor immune microenvironment
APCs: Antigen-presenting cells
TCR: T-cell receptor
TILs: Tumor-infiltrating lymphocytes
WES: Whole exome sequencing

## References

[1] Fassnacht M, Johanssen S, Quinkler M, Bucsky P, Hs Willenberg F, Beuschlein MT (2009). Limited prognostic value of the 2004 international union against cancer staging classification for adrenocortical carcinoma: proposal for a revised TNM classification. Cancer.

[2] Tierney J, Sv Chivukula J, Poirier SP, Schadde E, Hertl M, Kebebew E (2019). National Treatment Practice for adrenocortical carcinoma: have they changed and have we made any Progress?. J Clin Endocrinol Metab.

[3] Lacroix A (2010). Approach to the patient with adrenocortical carcinoma. J Clin Endocrinol Metab.

[4] Fassnacht M, Terzolo M, Allolio B, Baudin E, Haak H, Berruti A (2012). Combination chemotherapy in advanced adrenocortical carcinoma. N Engl J Med.

[5] Tripathi A, Plimack ER (2018). Immunotherapy for urothelial carcinoma: current evidence and future directions. Curr Urol Rep.

[6] Naing A, Meric-Bernstam F, Stephen B, Dd K, Hajjar J, Rodon Ahnert J, et al. Phase 2 study of pembrolizumab in patients with advanced rare cancers. J Immunother Cancer. 2020; 8(1): e000347. 10.1136/jitc-2019-000347 PMC7078933 32188704

[7] Jd Fumet C, Truntzer MY, Ghiringhelli F (1990). Tumour mutational burden as a biomarker for immunotherapy: current data and emerging concepts. Eur J Cancer (Oxford England).

[8] Hellmann MD, Ciuleanu TE, Pluzanski A, Lee JS, Otterson GA, Audigier-Valette C (2018). Nivolumab plus Ipilimumab in lung cancer with a high tumor mutational burden. N Engl J Med.

[9] Valero C, Lee M, Hoen D, Zehir A, Mf B, Ve S, et al. Response rates to anti-PD-1 immunotherapy in microsatellite-stable solid tumors with 10 or more mutations per Megabase. JAMA Oncol. 2021;7(5):739–43. 10.1001/jamaoncol.2020.7684 PMC7893543 33599686

[10] Mayakonda A, Lin D, Assenov Y, Plass C, Koeffler H (2018). Maftools: efficient and comprehensive analysis of somatic variants in cancer. Genome Res.

[11] Chan TA, Yarchoan M, Jaffee E, et al. Development of tumor mutation burden as an immunotherapy biomarker: utility for the oncology clinic. Ann Oncol. 2019;30(1):44–56. 10.1093/annonc/mdy495 PMC6336005 30395155

[12] Chalmers Z, Cf Connelly D, Fabrizio LG, Sm Ali R, Ennis AS (2017). Analysis of 100,000 human cancer genomes reveals the landscape of tumor mutational burden. Genome Med.

[13] Wu MC, Lin X (2009). Prior biological knowledge-based approaches for the analysis of genome-wide expression profiles using gene sets and pathways. Stat Methods Med Res.

[14] Chen B, Khodadoust M, Liu CL, Newman A, Alizadeh A (2018). Profiling tumor infiltrating immune cells with CIBERSORT. Methods Mol Biol (Clifton, NJ).

[15] Newman A, Liu C, Green M, Gentles A, Feng W, Xu Y, Cd H (2015). Robust enumeration of cell subsets from tissue expression profiles. Nat Methods.

[16] Taiwen L, Jingyu F, Wang B, Nicole T, Qianming C, Liu Jun S, Bo L (2017). TIMER: a web server for comprehensive analysis of tumor-infiltrating immune cells. Cancer Res.

[17] Jiang P, Gu S, Pan D, Fu J, Sahu A, Hu X (2018). Signatures of T cell dysfunction and exclusion predict cancer immunotherapy response. Nat Med.

[18] Nguyen L, Martens JWM, Van Hoeck A, Cuppen E (2020). Pan-cancer landscape of homologous recombination deficiency. Nat Commun.

[19] Kanapathipillai M (2018). Treating p53 mutant aggregation-associated cancer. Cancers (Basel).

[20] Han HS, Kwon YJ, Park SH, Kim EJ, Rho YS, Sin HS (2004). Potent effect of 5-HPBR, a butanoate derivative of 4-HPR, on cell growth and apoptosis in cancer cells. Int J Cancer.

[21] Silva CL, Olival A, Perestrelo R, Silva P, Tomás H, Câmara JS (2019). Untargeted urinary (1) H NMR-based Metabolomic pattern as a potential platform in breast cancer detection. Metabolites.

[22] Frezza C (2018). Histidine metabolism boosts cancer therapy. Nature.

[23] Li T, Apte U (2015). Bile acid metabolism and signaling in cholestasis, inflammation, and cancer. Adv Pharmacol.

[24] Kühn T, Stepien M, López-Nogueroles M, Damms-Machado A, Sookthai D, Johnson T (2020). Prediagnostic plasma bile acid levels and colon cancer risk: a prospective study. J Natl Cancer Inst.

[25] Yu C, You M, Zhang P, Zhang S, Yin Y, Zhang X (2021). A five-gene signature is a prognostic biomarker in pan-cancer and related with immunologically associated extracellular matrix. Cancer Med.

[26] Sánchez-Paulete AR, Teijeira A, Cueto FJ, Garasa S, Pérez-Gracia JL, Sánchez-Arráez A (2017). Antigen cross-presentation and T-cell cross-priming in cancer immunology and immunotherapy. Ann Oncol.

[27] Wilky BA (2019). Immune checkpoint inhibitors: the linchpins of modern immunotherapy. Immunol Rev.

[28] Ilyinskii PO, Kovalev GI, O'neil CP, Roy CJ, Michaud AM, Drefs NM (2018). Synthetic vaccine particles for durable cytolytic T lymphocyte responses and anti-tumor immunotherapy. PLoS One.

[29] Li B, Chan HL, Chen P (2019). Immune checkpoint inhibitors: basics and challenges. Curr Med Chem.

[30] Billon E, Finetti P, Bertucci A, Niccoli P, Birnbaum D, Mamessier E (2019). PDL1 expression is associated with longer postoperative, survival in adrenocortical carcinoma. Oncoimmunology.

[31] Patel SP, Kurzrock R (2015). PD-L1 expression as a predictive biomarker in cancer immunotherapy. Mol Cancer Ther.

[32] Am G, Kato S, Bazhenova L, Sp P, Gm F, Miller V (2017). Tumor mutational burden as an independent predictor of response to immunotherapy in diverse cancers. Mol Cancer Ther.

[33] Yang X, Shi J, Chen X, Jiang Y, Zhao H (2020). Efficacy of Cabozantinib and Nivolumab in treating hepatocellular carcinoma with RET amplification, high tumor mutational burden, and PD-L1 expression. Oncologist.

[34] Wei SC, Duffy CR, Allison JP (2018). Fundamental mechanisms of immune checkpoint blockade therapy. Cancer Discov.

[35] Gibney GT, Weiner LM, Atkins MB (2016). Predictive biomarkers for checkpoint inhibitor-based immunotherapy. Lancet Oncol.

[36] Massard C, Gordon MS, Sharma S, Rafii S, Wainberg ZA, Luke J (2016). Safety and efficacy of Durvalumab (MEDI4736), an anti-programmed cell death Ligand-1 immune checkpoint inhibitor, in patients with advanced urothelial bladder cancer. J Clin Oncol.

[37] Binnewies M, Roberts EW, Kersten K, Chan V, Fearon DF, Merad M (2018). Understanding the tumor immune microenvironment (TIME) for effective therapy. Nat Med.

[38] Farhood B, Najafi M, Mortezaee K (2019). CD8(+) cytotoxic T lymphocytes in cancer immunotherapy: a review. J Cell Physiol.

[39] Stenzinger A, Jd A, Maas J, Md S, Dm M, Mm W (2019). Tumor mutational burden standardization initiatives: recommendations for consistent tumor mutational burden assessment in clinical samples to guide immunotherapy treatment decisions. Gen Chromosomes Cancer.

[40] Fehrenbacher L, Spira A, Ballinger M, Kowanetz M, Vansteenkiste J, Mazieres J (2016). Atezolizumab versus docetaxel for patients with previously treated non-small-cell lung cancer (POPLAR): a multicentre, open-label, phase 2 randomised controlled trial. Lancet.

[41] Chalabi M, Cardona A, Nagarkar DR, Dhawahir SA, Gandara DR, Rittmeyer A (2020). Efficacy of chemotherapy and atezolizumab in patients with non-small-cell lung cancer receiving antibiotics and proton pump inhibitors: pooled post hoc analyses of the OAK and POPLAR trials. Ann Oncol.

[42] Landwehr LS, Altieri B, Schreiner J, Sbiera I, Weigand I, Kroiss M (2020). Interplay between glucocorticoids and tumor-infiltrating lymphocytes on the prognosis of adrenocortical carcinoma. J Immunother Cancer.

[43] Ragazzon B, Libé R, Gaujoux S, Assié G, Fratticci A, Launay P (2010). Transcriptome analysis reveals that p53 and {beta}-catenin alterations occur in a group of aggressive adrenocortical cancers. Cancer Res.

[44] Liu S, Ding G, Zhou Z, Feng C (2018). β-Catenin-driven adrenocortical carcinoma is characterized with immune exclusion. OncoTargets Ther.

[45] Hwang WL, Wolfson RL, Niemierko A, Marcus KJ, Dubois SG, Haas-Kogan D (2019). Clinical impact of tumor mutational burden in neuroblastoma. J Natl Cancer Inst.

[46] Wang L, Ge J, Lan Y, Shi Y, Luo Y, Tan Y (2020). Tumor mutational burden is associated with poor outcomes in diffuse glioma. BMC Cancer.

[47] Devarakonda S, Rotolo F, Tsao MS, Lanc I, Brambilla E, Masood A (2018). Tumor mutation burden as a biomarker in resected non-small-cell lung cancer. J Clin Oncol.

[48] Fumet JD, Truntzer C, Yarchoan M, Ghiringhelli F (2020). Tumour mutational burden as a biomarker for immunotherapy: current data and emerging concepts. Eur J Cancer.

[49] Samstein RM, Lee CH, Shoushtari AN, Hellmann MD, Shen R, Janjigian YY (2019). Tumor mutational load predicts survival after immunotherapy across multiple cancer types. Nat Genet.

[50] Jia Q, Wang J, He N, He J, Zhu B (2019). Titin mutation associated with responsiveness to checkpoint blockades in solid tumors. JCI Insight.

[51] Li X, Pasche B, Zhang W, Chen K (2018). Association of MUC16 mutation with tumor mutation load and outcomes in patients with gastric cancer. JAMA Oncol.

[52] Zhang L, Han X, Shi Y (2020). Association of MUC16 mutation with response to immune checkpoint inhibitors in solid tumors. JAMA Netw Open.

[53] Felder M, Kapur A, Gonzalez-Bosquet J, Horibata S, Heintz J, Albrecht R, Fass L (2014). MUC16 (CA125): tumor biomarker to cancer therapy, a work in progress. Mol Cancer.

